# The Year-Book of Treatment for 1884

**Published:** 1885-06

**Authors:** 


**The Year-Book of Treatment for 1884.** A Critical Review for Practitioners of Medicine and Surgery. 8vo., p.p. 308. Philadelphia: Lea Brothers & Co. 1885.

The preface states that "the object of this book is to present to the practitioner not only a complete account of all the more important advances made in the treatment of disease, but to furnish also a review of the same by competent authorities." The value of such a work will be apparent to all at this day, when medical literature is teeming with innumerable so-called advances. The resumé has been made by a number of well-known masters in special fields of medicine and surgery, and, after carefully looking over the work, we can say that the object stated in the preface has been fairly attained. The practitioner will find here recorded all the more important advances, relating to treatment, that have been published during the year ending September 30, 1884.

